# Improving the longevity of optically-read quantum dot physical unclonable functions

**DOI:** 10.1038/s41598-021-90129-2

**Published:** 2021-05-26

**Authors:** Kieran D. Longmate, Nema M. Abdelazim, Elliott M. Ball, Joonas Majaniemi, Robert J. Young

**Affiliations:** 1 Physics Department, Lancaster University, Lancaster, LA1 4YB UK; 2 School of Electronics and Computer Science, University of Southampton, Southampton, SO17 1BJ UK

**Keywords:** Chemistry, Mathematics and computing, Nanoscience and technology, Physics

## Abstract

Quantum dot physically unclonable functions (QD-PUFs) provide a promising solution to the issue of counterfeiting. When quantum dots are deposited on a surface to create a token, they form a unique pattern that is unlikely to ever be reproduced in another token that is manufactured using the same process. It would also be an extreme engineering challenge to deterministically place quantum dots to create a forgery of a specific device. The degradation of the optical response of quantum dots over time, however, places a limitation on their practical usefulness. Here we report methods to minimise the degradation of photoluminescence (PL) from InP/ZnS quantum dots suspended in a polymer and demonstrate reliable authentication using a fingerprinting technique to extract a signature from PL, even after significant degradation has occurred. Using these techniques, it was found that the addition of a polylauryl methacrylate (PLMA) copolymer improved the longevity of devices. The best performing example of this was the Polystyrene-PLMA based material. From this, it is projected that 1000 bits of information could be extracted and read after a period of years, therefore providing a compelling solution to the issue of counterfeiting.

## Introduction

Each year billions of pounds are lost due to ineffective anti-counterfeiting measures^1^. Many contemporary anti-counterfeiting solutions rely on simply being difficult to replicate, often restricting access to materials or through complex patterns^2,3^. The key issue is one of a lack of asymmetry in the anti-counterfeiting measure’s production. There is nothing that physically prevents an attacker from cloning some of the most complex anti-counterfeiting measures^4^.

One method of solving this issue can be found in physically unclonable functions (PUFs)^5,6^. A PUF is a hardware-based cryptographic primitive which, in a perfect scenario, provides a unique fingerprint for authentication purposes. This information will only become accessible to a user as a response to a particular challenge^5^. From a cryptographic standpoint, PUFs are classified as one-way functions, their unique nature prevents an attacker from deciphering how to recreate a particular PUF^4^. Methods of creating PUFs include stochastic processes^7^ and nanomaterials^8^, in particular optically-active quantum dots^1,9^.

Colloidal quantum dots (QDs) suspended in a lacquer or solvent and applied to a substrate prior to curing, can form a token containing a random arrangement of dots, formed in clusters with a range of sizes^10^. Owing to the effect that quantum dot clustering has on the energy levels within each CQD this produces a unique emission pattern when excited. This by itself however, can be simulated. The appeal of CQDs therefore lies in their non-linear response to increasing incident light, as this can be used to detect the presence of a fake^1,11^. Thus, CQD patterns can be used for authentication purposes.

Here we demonstrate the creation of such optical-read tokens known as quantum dot PUFs (QD-PUFs). A response derived from dots’ emission in the token is stable for a finite period of time, which is tested using an authentication algorithm. Emission from the tokens is excited using incoherent light within the visible spectrum.

The stability of emission from colloidal QDs (CQDs) depends on the respective stability of their morphology and chemistry, which is typically limited by the rate of oxidization when they are exposed to air^12^. To reduce this, CQDs can be suspended in a polymer matrix, where the effectiveness of this approach depends on the polymer’s oxygen diffusion rate and their compatibility with the CQDs used^13^. As such alongside the creation of the QD-PUF token five different polymers (PMMA, PS, PMS, PVDF and SEBS), and their co-polymer variants when combined with PLMA, were tested to maximise longevity and emission intensity.

## Experimental

To create the QD-PUF tokens, InP/ZnS QDs with oleylamine surface ligands were dissolved in toluene before being combined with a polymer. This solution was then applied to a black coloured polyethene substrate using a micrometre doctor-blade method to form a dry film with fixed deposition thickness. The tags were then kept at 60 °C in a vacuum oven overnight to cure the polymer. Specific details about the parameters used can be found in the Supplementary Information. Each QD-PUF token was then stored in a container that was open to the air. This ensured that the only factor mitigating the degradation of the emission from the CQDs was the lacquer they were suspended in.

To determine the optimal polymer matrix to minimise oxidization of the QDs suspended within, five separate polymers were tested initially [Polymethyl methacrylate (PMMA), Polystyrene (PS), Polystyrene-ethylene-butylene-styrene (SEBS), polyvinylidene fluoride (PVDF) and Poly 4-methlstyene (PMS)], which are labelled Group 1 in this work.

The addition to the formulations in Group 1, a second test group of tokens (dubbed Group 2) was created with the addition of polylauryl methacrylate (PLMA) to the five polymers from Group 1. This acted as a copolymer and was added to achieve more rapid photoluminescence stabilization.

**Figure 1:**
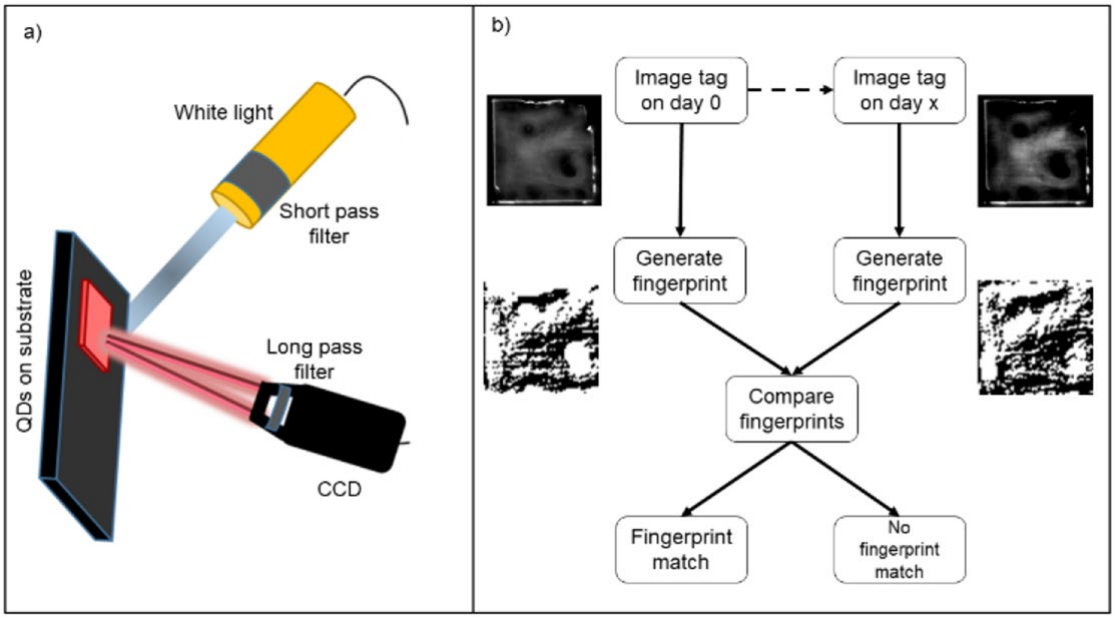
(**a**) A schematic of the apparatus used to measure PL intensity maps and to captures images of each token. The quantum dots in each token were excited using white light filtered through a 450 nm short-pass filter. A 500 nm long-pass filter placed in front of the CCD ensures only light from the emission of the QD-PUF is measured. The entire apparatus is sealed within a closed black box when measurements are in progress. (**b**) Using the apparatus in (**a**) each token is imaged on the day of its creation and each subsequent day after. The fingerprint generated on the subsequent days is compared to that of day 0 to determine if it matches the original fingerprint.

Photoluminescence (PL) from each token was measured using the same apparatus as was used to apply the challenge to extract the fingerprint. This is shown in Fig. 1a. This approach has benefits over previous work^1^ involving QD-PUFs, as there is no requirement for high-resolution optics to extract responses from the tokens.

For the purpose of turning the captured images of the PL emission patterns from each token into a binary response for a computer vision technique known as Local Binary Patterns (LBP)^14,15^ was applied in a modified form. The procedure used is shown in Fig. 1b and detailed in the Supplementary Information. This modified variant was used to generate noise-resistant fingerprints and is referred to as the reduced LBP (R-LBP). In-depth analysis of the cryptographic security of the generated fingerprints is beyond the scope of this paper which demonstrates them as a proof of concept. The metrics used to analyse the R-LBP results (FPR and ENIB) serve as a measure to judge the stability of QD-PUF generated fingerprints over time. The false negative rates (FNR) of the generated fingerprints are not reported as their value fell below the precision of the implementation of the code used in MathWorks MATLAB.

After having been made (day 0), each token was imaged 100 times, and R-LBP algorithm was applied to each capture to generate a corresponding fingerprint. 50 of these fingerprints were set aside to use as reference fingerprints in order to calculate hamming distances later on. The other 50 were used as test fingerprints. Each test fingerprint was compared to each of the reference fingerprints of the same tag, in order to generate an intra-hamming distance distribution. The test fingerprints were then compared to each reference fingerprint of every other token type in order to generate the inter-hamming distribution. On each subsequent day, 50 further images of each token were taken and used to generate test fingerprints. Each of these was then compared to the day 0 reference fingerprints, in the same manner, to generate hamming distance distributions. From the overlap of the hamming distance distributions, the false positive rate (FPR) of each token on each day was calculated.

The FPR provides a quantitative measure of a fingerprint’s uniqueness. The FPR of a fingerprint determines the probability of another fingerprint being found that matches the one being tested. It is calculated by taking the overlap between the inter- and intra-hamming distance distributions. This is performed by calculating the probability that an element of the inter hamming distance distribution falls below the maximum element of the intra distribution. It also serves as a metric of the possibility of the fingerprint being reproduced. Thus, providing a measure of the effectiveness of the tokens as a long-term anti-counterfeiting solution.

## Results

### Group 1 results

**Figure 2:**
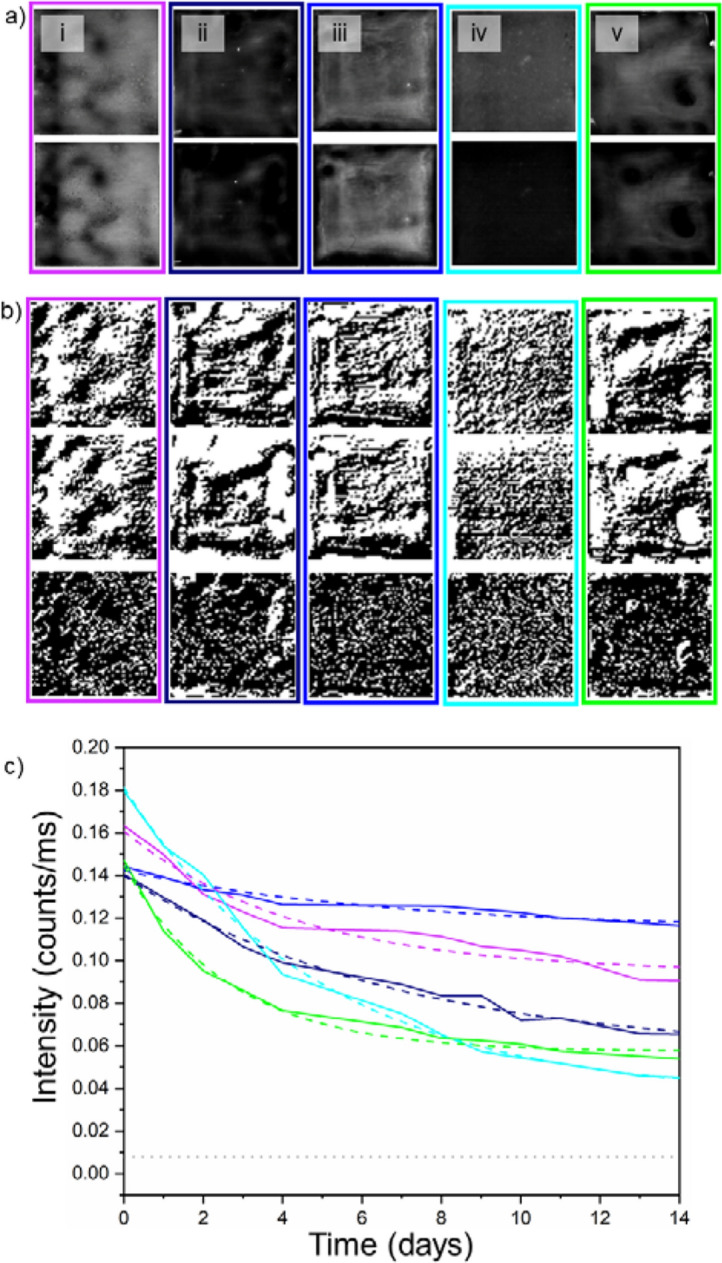
(a) ﻿Top—Images showing photoluminescence (PL) intensity maps from each formulation of the Group 1 tokens captured on the day they were created (day 0). Bottom—PL images of each Group 1 token type captured on day 14. The intensity of day 14 images has been increased by 30% post-capture to aid visual comparison. (i) PMMA, (ii) PMS, (iii) PS, (iv) PVDF, (v) SEBS. (**b**) The top of each coloured rectangle is the R-LBP generated fingerprint of the labelled QD-PUF on day 0. The middle of each coloured rectangle is the fingerprint from day 14. In the bottom image of each rectangle, the white pixels indicate the pixels that changed in value in the fingerprint between day 1 and 14. The percentage of pixels that changed value and the composition of each is as follows: (i) PMMA, 21.2%, (ii) PMS, 24.5%, (iii) PS, 18.9%, (iv) PVDF, 29.1% (v) SEBS, 19.3%. (**c**) A plot showing the average PL intensity from each token type as a function of time since their creation. An exponential decay fit was applied to each (dashed line). The grey dotted line represents the background noise signal from the CCD-sensor.

Figure 2a shows the PL emission intensity maps measured from each token type as a function of time since their creation. Between measurements, the tokens were stored in ambient laboratory conditions, exposed to air and moisture. Figure 2b displays the binary fingerprints generated from each token on day 0 alongside what the fingerprint of the same token looked like on day 14. Figure 2c plots the average intensity from each token type, and clearly shows their degradation as they aged, with the effect being more pronounced for some encapsulating polymers than others.

Owing to the oxidation mechanism responsible for the degradation of PL from the CQDs^12^ an exponential decay fit of the form, y = l_min_ + A × exp(R_0_ × x) (where l_min_ represents the asymptote of the PL curve and R_0_ the decay constant) was applied to extrapolate the anticipated emission intensity following stabilization after a long period of time. A key metric for practical consideration, is to ensure that the emission intensity from the tokens is above the background noise level measured by the CCD sensor—to ensure facile measurement of the signal. While this provides no measure of the token’s performance in a daylight level environment, it does ensure that the tokens are still emitting to a degree that can be measured. Stability tests from Group 1 samples revealed that, among the five types of polymer, PS showed the greatest longevity, likely due to resisting oxidization, losing 19.6% of its original intensity over several days before stabilizing. The key to the token’s stability is the oxygen diffusion rate of the polymer that the dots are embedded in Ref.^13^. For example, SEBS triblock copolymer has higher oxygen diffusion rate than the PS block^16^. It was found that the stability depended on the type of the polymer host matrix. For example, when PVDF was used to create the token, the PL intensity rapidly decayed (losing > 80% of its intensity in a few days before stabilizing). In comparison, tokens created with PS show a much lower decay rate over the period measured. Use of a matched polymer minimizes ligand loss in the encapsulation process and minimizes oxygen diffusion to the QD surface^16^. As the lowest asymptotes for the curves fitted to the experimental data (for PVDF) was found to be 4.5 times that of the background counts, it is clear that all of the polymers provide sufficient stability for tokens created with them to be measured for the feasible lifetime of a typical anticounterfeiting device, i.e. several years.

**Figure 3:**
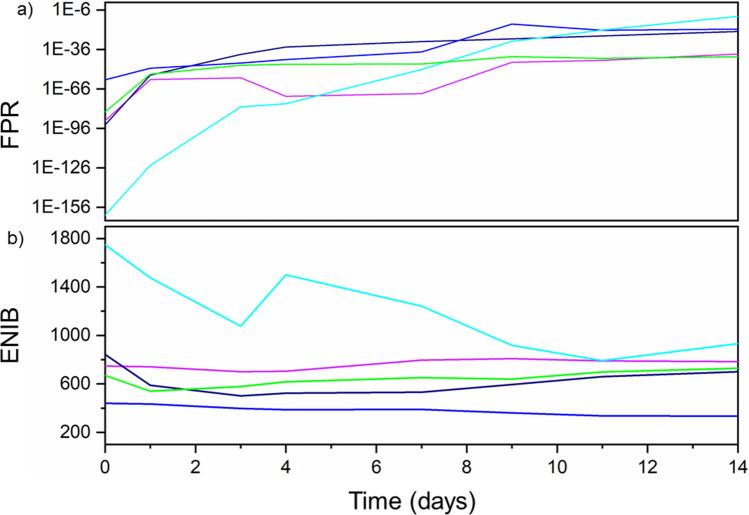
Top—A plot showing the false positive rate (FPR) of each Group 1 token type, which was derived by comparing fingerprints generated from photoluminescence images captured at the times shown after their creation. Bottom—Showing the effective number of independent bits (ENIB) extracted from each Group 1 token as a function of days since their creation.

Figure 2b shows the fingerprints generated on the first and the last day of the trial for each token type, as well as a pixel map comparing the difference between them. This gives both a visual representation of the fingerprint’s uniqueness and a quantitative measure of how the fingerprints extracted from the tokens decay over time. As can be seen in Fig. 3a each of the fingerprints do have an initial FPR that renders the probability of a forgery being accepted as negligible. Although the reference images were taken at the same time as the test images, noise within each image renders this value non-zero. What is of particular interest is how the FPR changes over time. Each of the FPR data sets fit an exponential decay model. The origin of this can be traced back to the PL intensity measured from each token, which decreases exponentially. The increasing influence of noise as the signal–noise ratio reduces and leads to an increasing FPR. This indicates that in order for a generated fingerprint to be stable over time, the emission from the dots in the token should also be stable.

The effective number of independent bits (ENIB) of a particular fingerprint determines the maximum length of an authentication key that can be extracted from that fingerprint^17^. Not all bits can be used as certain patterns, and trends are repeated over multiple fingerprints. Although it is often standard to discuss entropy for authentication metrics in the case of this experiment we are interested in the potential applications of such fingerprints. Many applications of such a concept require a minimum number of bits, thus making ENIB a more informative metric. To discuss the ENIB, it is best to separate the Group 1 tokens, as the PVDF-based token performs differently from the rest. With the exception of PVDF, there is no discernible relationship between decreasing PL intensity over time and the measured ENIB, as shown in the of Fig. 3b. Despite decreases in the PL intensity, the ENIB remains roughly constant. Furthermore, this removes a possible link between the initial PL intensity and a token’s starting ENIB. If the two were indeed dependant on each other, a dependence between PL intensity and ENIB would be seen at later days. This, therefore, implies that the ENIB is instead dependent on the arrangement of the QD pattern and the PL intensity stays sufficiently intense that measured noise causes no degradation in the extracted ENIB during the course of this trial. This provides further insight into why PVDF has the highest ENIB and has the only ENIB with a discernible decrease in value with time. Unlike the other token types, PVDF does not show a clustering effect in its pattern; it is more uniform in its distribution. Its lack of a clearly defined structure means that it is more susceptible to noise, which is not an issue when there is a high signal–noise ratio. As this ratio reduces, however, PVDF’s ENIB drops as the structures in its PL intensity map become more difficult to discern. The other token types, the PL images from which show more clustered patterning, display similar ENIB values that do not decay significantly over time, despite the decreasing signal–noise ratio.

### Group 2 results

The aim of the creating the tokens in Group 2 was to study to influence of combining two polymers to encapsulate the dots; using a block co-polymer (PLMA) to enhance the stability of emission from the dots in the token. Unlike the polymers used in Group 1, PLMA is a high viscous oily liquid that is hydrophobic^18^, with its elasticity making it suitable for use as a block copolymer to the main polymers used in the Group 1 tokens. Alongside this, the addition of PLMA was found to increase the interface angle of the surface and/or dot aggregates, which are capable of being used to produce complex identifiers and are more difficult to duplicate^19^.

**Figure 4:**
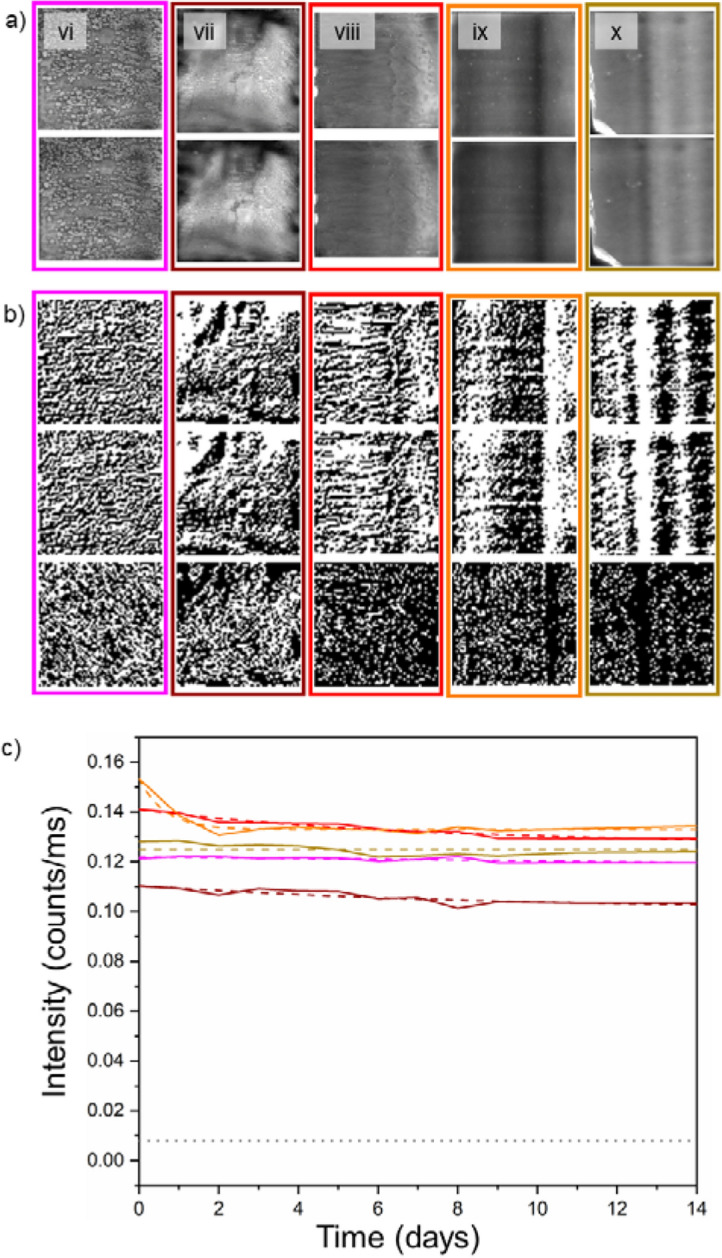
(**a**) Top—Images showing the photoluminescence (PL) intensity maps from each formulation of the Group 2 tokens captured on the day they were created (day 0). Bottom—PL images of each Group 1 token type captured on day 14. The intensity of the day 14 image has not been increased post capture, as it was in Fig. 2. For each token type the polymer used was: (vi) PMMAPLMA, (vii) PMSPLMA, (viii) PSPLMA, (ix) PVDFPLMA, (x) SEBSPLMA. (**b**) The top of each coloured rectangle is the R-LBP generated fingerprint of the labelled QD-PUF on day 0. The middle of each coloured rectangle is the fingerprint from day 14. In the bottom image of each rectangle, the white pixels indicate the pixels that changed in value in the fingerprint between day 1 and 14. The percentage of pixels that changed value and the composition of each is as follows: (vi) PMMAPLMA, 46.5%, (vii) PMSPLMA, 41.2%, (viii) PSPLMA, 19.5%, (ix) PVDFPLMA, 24.12%, (x) SEBSPLMA, 15.4%. (**c**) The average PL intensity from each token type as a function of time since their creation. An exponential decay fit was applied to each (dashed line). The grey dotted line represents the background noise signal from the CCD sensor.

Figure 4c shows that token type in Group 2 with the fastest temporal decay (PVDFPLMA with a 13.0% drop) outperforms the slowest decaying token type in Group 1 (PS with a 19.6% drop). This indicates that the viscous copolymer is protecting the dots from degradation, likely by blocking ambient oxygen from reaching their surface^16^. It is interesting to note, however, that PVDF containing lacquer is once again the worst-performing. This would suggest a lack of compatibility between PVDF and the quantum dots^13^.

Finally, on the topic of PL is the matter of the long-term viability of utilizing the token’s emission as a PUF. Once again, the data in Fig. 4c has been fit to an exponential decay model. This shows that the PL from the token stabilizes rapidly and emits with an intensity that is significantly above the noise floor of this experiment for a long period of time. In the case of the Group 2 tokens, the lowest asymptotic value for the PL intensity is for the PMSPLMA based token, which is 12.5 times greater than the background level. This shows that all of the token types in Group 2 are indeed suitable for use as PUFs in applications that require emission to be visible after a long period of time.

**Figure 5:**
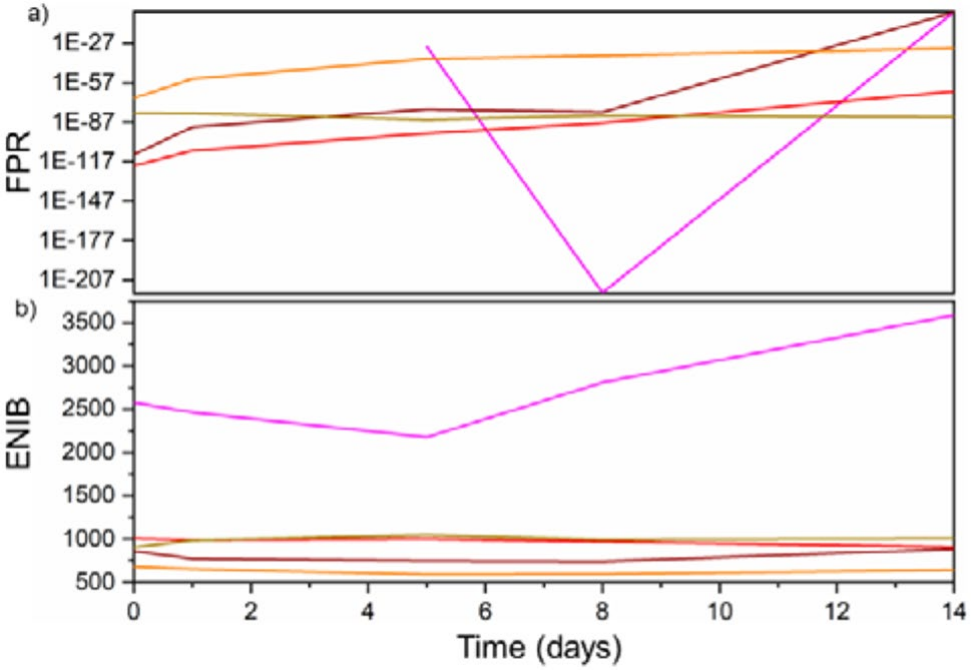
Top—Showing the false positive rate (FPR) of each token type in Group 2, which was derived by comparing fingerprints generated from photoluminescence captured at the times shown after their creation. Token vi displays no FPR before Day 5 as its value fell below the precision of the implementation of the code used MathWorks MATLAB. Bottom—showing the effective number of independent bits extracted from each Group 2 token as a function of days since their creation.

As with the previous group, Fig. 4b gives a visual representation of the generated fingerprints. It demonstrates that not only do the fingerprints bear a resemblance to the token they originated from (as can be seen from Fig. 4a) but that each is distinct from that of other tokens. With two exceptions (PMMAPLMA and PMSPLMA) each of the FPR curves shown in Fig. 5a neatly follows an exponential fit. Lending further support to the hypothesis that the FPR is dependent on the signal–noise ratio. The data of SEBSPLMA also lends credence to this. The shape of its PL curve is much flatter than the others, with the only fluctuations owing to noise, which is reflected in its FPR. PMMAPLMA, on the other hand shows data that varies greatly. Its FPR values before day 5 fell below the precision of the implementation of the code used in MathWorks MATLAB (namely 10^–308^) when the data was analysed and so were set to zero. After this PMMAPLMA’s FPR rapidly rises and falls again, despite having stable PL. This suggests that there is another influential factor the driving force behind this. The most likely reasoning behind this is the quantum dot pattern itself and its interpretation by the R-LBP algorithm, making the final fingerprint highly susceptible to noise^20^. This is also the likely reasoning behind PMSPLMA’s deviation from a smooth curve. It is hypothesized that there are optimal ranges of feature sizes for each radius of R-LBP, however further testing is needed to confirm this. The range of initial values achieved (2.6 × 10^–69^ to 7.1 × 10^–121^) once again renders the probability of a forgery being accepted as negligible. When compared to the Group 1 data, it is clear that the inclusion of a co-polymer improves the FPR. For PSPLMA, PVDFPLMA and SEBSPLMA, which all followed a smooth trend in their data, each showed an improvement on the day 14 value of their FPR when compared to their Group 1 counterpart. The origin of this stems from the greatly improved stability of the PL intensity that is found in the Group 2 set of tokens.

In regards to the ENIB of Group 2 the first matter which is apparent is that once again there appears to be no discernible link between the PL value of a token type and the ENIB of the fingerprint generated from it. As can be seen from comparison of Fig. 5a,b. This indicates that, once again, the value of the ENIB is solely dependent on the pattern of the emission from the dots itself. In this case, however as the patterns cannot be separated out into distinct groups based on their type of pattern, no conclusions can be drawn as to which patterns benefit ENIB the most. To do this, more stringent criteria to define the different types of quantum dot patterns are needed. One approach to this would be based on a histogram of feature sizes and could be performed through the non-modified version of LBP.

## Concluding remarks

By measuring PL emission from colloidal InP/ZnS quantum dots suspended in a polymer film, and applying a modified version of local binary pattern algorithm, tokens were created that can be used as physically unclonable functions in anti-counterfeiting applications. The response from these tokens takes the form of a 64 × 64 matrix of bits, in which information is derived from pattern quantum dot clusters that are locked in the polymer when it was cured. PL from each of the token formulations tested stabilized at a value above the background noise floor of our apparatus. As the FPR of each fingerprint was found to be dependent on the signal–noise ratio of the image it was generated from, stabilization of the emission from the token resulted in a respective stabilization in the measured FPR. Although it is shown that both groups of token types are stable, the addition of PLMA copolymer in the token’s formulation significantly reduced temporal decay of the PL intensity and FPR.

By applying a modified version of the LBP algorithm, it possible to preserve a unique fingerprint generated from the optical response of a token, even after substantial degradation. In the worst-case scenario, for the token types with temporal FPR dependencies that tended towards an asymptote (e.g. for PVDF in Group 1), the probability of a tag incorrectly identifying positively remained below one in a million. This ensures that the only methods of copying a token, is to either replicate its fingerprint digitally or by brute force guesswork. When coupled with the lack of temporal decay in the ENIB, this ensures that each token type (with the exception of PS in Group 1) would be able to be used as secure authentication primitive for purposes requiring at least 512-bit keys.

The security of QD-PUFs lies in their unique nature, and as such, any trends in the patterning that occur in the emission from different tokens is detrimental to this. Future analysis of large batches of the most promising token types must be carried out to investigate this. Whether these be ensuring no manufacturing artefacts are formed on the QD pattern or introducing more entropy to the fingerprints. A method to achieve this could be to use the fingerprint as the input for a fuzzy extractor^21^. This would take the non-uniformly random fingerprint and use it as a seed for a uniformly random bit string. Thus, removing the effect of any trends while maintaining stability.

## Supplementary Information

Supplementary Information.
