# Supplementary material for: Improving the longevity of optically-read quantum dot physical unclonable functions
Source: Sci Rep. 2021 May 26;11:10999. doi: 10.1038/s41598-021-90129-2 (PMC8155066; doi:10.1038/s41598-021-90129-2)
Supplement: Supplementary file 1 — Supplementary Information. [file 41598_2021_90129_MOESM1_ESM.pdf]

Improving the longevity of optically-read quantum dot physical unclonable functions

Kieran D. Longmate[1], Nema M. Abdelazim[1,2], Elliott M. Ball[1], Joonas Majaniemi[1] & Robert J. Young[1*]

1. *Physics Department, Lancaster University, Lancaster LA1 4YB, U.K.*
2. *School of Electronics and Computer Science, University of Southampton, Southampton SO17 1BJ, U.K.*

Supplementary Information

The reduced local binary pattern (R-LBP) algorithm

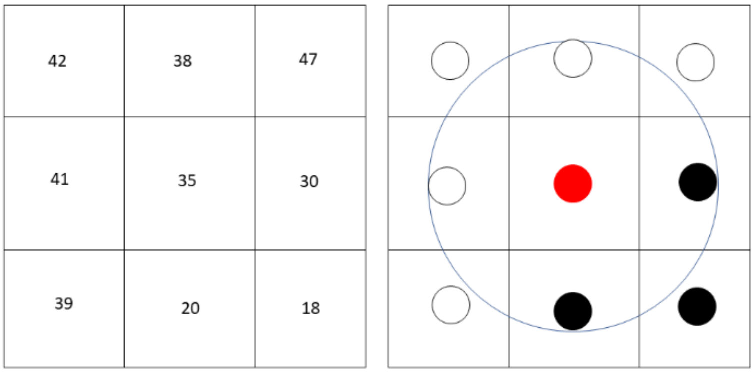

Figure 1: Left: an example cell array with cell values. Right: the application of LBP to the application of LBP to this cell array. The red circle represents the pixel being analysed. White circles represent a pixel assigned the value of 1, black those assigned the value of 0.

The initial framework for the Local Binary Pattern (LBP) code originated from the University of Oulu's CMV group [1] .The function they used to apply the LBP fingerprinting algorithm was more detailed and simpler to modify for the specific needs required here than MATLAB's built-in LBP function. How LBP operates is covered in Figure 1, the driving principle behind it is the compiling of local information via pixel comparison [2]. LBP does this by moving across a greyscale image on a pixel-wise basis. At each pixel, it compares the values of the pixels that lie on the circumference of a circle (whose radius is fixed and predetermined) centred on that pixel, to that of the centre pixel. If a pixel on the circumference is greater in value than the centre pixel, it is assigned a value of 1; otherwise, it is assigned a value of 0. The pixels' values on the circumference are then concatenated into a single binary string, starting at the top and moving clockwise. This string is then assigned to the central pixel, and the code moves onto the next pixel. This variant of LBP is known as reduced LBP (R-LBP).

The arrangement of the binary values provides information about the local area of the central pixel. From this, one can determine if the central pixel sits on an edge, a corner or other image feature [3]. In standard LBP each of the binary strings would be compiled into a feature histogram, to allow the computer to determine what features are present in an image. This, however, does not provide a key that can be authenticated. Using every bit in the strings assigned to a pixel proved too noisy to be of use. As such, to create a stable authentication key using LBP information had to be sacrificed. Before the binary string is assigned to the central pixel, the string's weighted modal value is found. This is performed by determining if the number of pixels with the value of 1 is greater than a predetermined threshold (usually set to 0.5). If this is the case, the central pixel is assigned the value of 1. If not, it is assigned the value of 0. While sacrificing the local information of the pixel, this averaging allows for the creation of a binary fingerprint for authentication purposes, which possesses robustness against the effects of image noise. This removes the ability to discern LBP features, but the trade-off for a stable, global illumination invariant binary fingerprint makes this acceptable. Not only this but the adaptable nature of LBP, as well as the many variants available, allow for easy improvements to the binary fingerprint. For example, the introduction of rotation

invariance, blur reduction by merging with Histogram-Oriented Gradients (HOG) or the inclusion of information on local gradients.

QD-PUF token creation

Controlling the quantum dots' dispersion in a particular polymer matrix is crucial to preserving their physicochemical properties for their application. We compared the stability of the QD/PNCs (polymer nanocomposites) containing different types of polymers.

Composites were formed by mixing solutions of QDs and hydrophobic–polymers in toluene. Various polymers that are transparent in the visible spectral range were chosen as the embedding matrix for the dots, to enhance the stability and longevity of the final token. The dot-polymer mixture was applied to black-coloured polyethene substrates using a micrometre doctor-blade, which forms a dry film with a fixed deposition thickness set to 5 µm. Six different hydrophobic polymers including Poly(methyl methacrylate) PMMA, Polystyrene PS, Poly(styrene-ethylene-butylene-styrene) SEBS, poly(vinylidene) fluoride PVDF, Poly(lauryl methacrylate) PLMA, and Poly 4-methlstyene PMS were used to produce the tokens, which were applied directly after the mixing procedure to the active area of the mount, prior to curing. These polymers were selected owing to their solubility in organic solvents, which match with the non-aqueous QDs used. The tokens were stored in ambient conditions and measurements of the relative PL emission intensities were made to gauge the stability of the formulations over the course of 2 weeks.

For the preparation of the token types in Group 1, InP/ZnS Colloidal Quantum Dots (CQDs) with oleylamine surface ligands were dissolved as a wet powder into 250 µl of toluene to form solution 1; the polymer was dissolved in toluene (0.1 g/ml) to obtain solution 2. Then two solutions were mixed with final concentrations of ~ 80 mg/ml by vigorously stirring them together, followed by sonication for 30 min to agitate them in the solution. This solution was applied to the polyethene substrate using the doctor blade, by placing 300 µl of the mixture to form a dry film applied to the substrate (mount active area) with a fixed deposition thickness set to 5 µm at room-temperature. The tags were then kept at 60° C in a vacuum oven overnight to ensure they fully cured.

The preparation of mixtures for the token types in Group 2 varied slightly to that of Group 1. The QDs were dissolved into the polymer to form a solution that was kept stirring for 1 hr, as described in Group 1 process. After this, the copolymer was added to give a final concentration of ~ 80 mg/ml. The ratio between of polymer:copolymer used was 95:5. The solution was stirred for 2 days before depositing it onto the substrate, following the procedure used to create the Group 1 tokens.

Bibliography

[1] “Matlab code,” Center for Machine Vision and Signal Analysis., 2015. [Online]. Available: http://www.cse.oulu.fi/CMV/Downloads/LBPMatlab.

[2] T. Ojala, M. Pietikained and D. Harwood, “A Comparative Study of Texture Measures with Classification Based on Feature Distributions,” *Pattern Recognition, ,* vol. 29, pp. 51-59, 1996.

[3] T. Maenpaa and M. Pietkainen, Texture Analysis with Local Binary Patterns. In *Handbook of Pattern Recognition and Computer Vision,* 197-216 (2005).
